# Lack of association of vitamin D receptor BsmI gene polymorphism with bone mineral density in Spanish postmenopausal women

**DOI:** 10.7717/peerj.953

**Published:** 2015-07-02

**Authors:** Jose M. Moran, Maria Pedrera-Canal, Francisco J. Rodriguez-Velasco, Vicente Vera, Jesus M. Lavado-Garcia, Pilar Fernandez, Juan D. Pedrera-Zamorano

**Affiliations:** Metabolic Bone Diseases Research Group, Facultad de Enfermería y Terapia Ocupacional, Universidad de Extremadura, Cáceres, Spain

**Keywords:** VDR polymorphism, BsmI, Postmenopausal, Osteoporosis

## Abstract

Osteoporosis is a polygenic disorder that is determined by the effects of several genes, each with relatively modest effects on bone mass. The aim of this study was to determine whether the vitamin D receptor single nucleotide polymorphism BsmI is associated with bone mineral density (BMD) in Spanish postmenopausal women. A total of 210 unrelated healthy postmenopausal women aged 60 ± 8 years were genotyped using TaqMan^®^ SNP Genotyping Assays. Lumbar and femoral BMD were determined by dual-energy X-ray absorptiometry (DEXA). Daily calcium and vitamin D intake were determined by a food questionnaire. No differences were found in the femoral neck, trochanter, Ward’s Triangle, L2, L3, L4, L2-L4, or between the femoral neck and total hip BMD after further adjustment for potential confounding factors (*P* > 0.05) (age, BMI, years since menopause and daily calcium intake). The BsmI polymorphism in the VDR gene was not associated with BMD in Spanish postmenopausal women.

## Introduction

Osteoporosis is a common disease characterized by low bone mass, disturbed microarchitecture of the bone tissue, and increased fracture risk. Osteoporosis is defined to exist when bone mineral density (BMD) values in the spine or hip fall 2.5 standard deviations (SD; T-score values) or more below the population average in young adults (Kanis et al., 2002). Peak bone mass is attained in early adult life but declines in postmenopausal women as a result of a reduction in estrogen production with effects on bone as well as intestinal and renal calcium handling (Prince & Dick, 1997).

Previous studies in twins and families showed that genetic factors play an important role in the formation of BMD and that genetic influence can account for up to 85% of the bone mass, with the strongest effects in the axial skeleton (Pocock et al., 1987; Christian et al., 1989; Slemenda et al., 1991).

Thus, osteoporosis is a polygenic disorder that is determined by the effects of several genes, each with relatively modest effects on bone mass and other determinants of fracture risk. Population-based studies and case-control studies have similarly identified polymorphisms in several candidate genes that have been associated with bone mass or osteoporotic fracture, including the vitamin D receptor (*VDR*) (Morrison et al., 1994), estrogen receptor (Kobayashi et al., 1996), regulator genes of the synthesis of TGF-*β*1 (Langdahl et al., 1997) and collagen type I *α*-1 gene (Grant et al., 1996). Vitamin D, through its principal bioactive form 1,25-dihydroxyvitamin D3 (1,25-(OH)2D3), plays a crucial role in bone metabolism. The action of 1,25-(OH)2D3 is mediated through a specific hormone receptor (Ralston & de Crombrugghe, 2006). Mutations in the *VDR* gene cause the syndrome of vitamin D-resistant rickets, which is a recessive condition characterized by alopecia, hypocalcaemia, hypophosphatemia, and severe rickets and is resistant to treatment with vitamin D and its active metabolites (Kristjansson et al., 1993; Ralston & de Crombrugghe, 2006). *VDR* was the first candidate gene to be studied in relation to osteoporosis (Morrison et al., 1994; corrected in 1997), and most attention has focused on polymorphisms situated near the 3′ end of *VDR* that are recognized by the restriction enzymes BsmI, ApaI and TaqI (Ralston & de Crombrugghe, 2006).

The discrepancies between studies addressing genetic risks may be attributed to genetic heterogeneity, population admixture and gene-environment or gene-gene interactions (Salanti, Sanderson & Higgins, 2005). Although many studies have been conducted, the results have given rise to a plethora of arguments on both sides of this issue (Uitterlinden et al., 2006; Ji et al., 2010). Hence, the results from numerous studies are inconclusive, and there is still no clear answer whether a specific *VDR* genotype is associated with lower BMD.

This study aims to investigate the relationship of a commonly studied polymorphism in the *VDR* gene intron 8, BsmI, with BMD in a cohort of Spanish postmenopausal women.

## Materials & Methods

### Study population

Participants were 210 unrelated healthy postmenopausal women 60 ± 8 years of age who were recruited voluntarily from February to December 2013. The group included 150 women with osteoporosis, 30 women with osteopenia and 30 healthy individuals. The study was performed in accordance with the Declaration of Helsinki and was approved by the Research Ethics Committee of the University of Extremadura. Written informed consent was obtained from all subjects.

### Densitometric study

Densitometric measurements were performed to define the BMD in the femoral neck (FN), femoral trochanter (FT), Ward’s triangle (WT) and the lumbar spine at the L2, L3, L4 and L2-L4 levels. Additionally, body weight and height were measured to calculate the body mass index (BMI). Densitometric tests were performed with the use of a NORLAND XR-800 (Norland Medical Systems, Inc., White Plains, New York, USA). BMD scores were expressed as grams per square centimeter.

### Laboratory analysis

The following SNP was included: rs1544410 (BsmI). SNP was evaluated by allelic discrimination real-time PCR using a TaqMan probe (Applied Biosystems, Foster City, California, USA). The PCR consisted of a hot start at 95 °C for 10 min followed by 40 cycles of 94 °C for 15 s and 60 °C for 1 min. Fluorescence detection occurred at a temperature of 60 °C. All assays were performed in 10-µl reactions using TaqMan Genotyping Master Mix in 48-well plates on a StepOne^®^ instrument (Applied Biosystems, Foster City, California, USA). Control samples representing all possible genotypes and a negative control were included in each reaction. The concordance of blinded quality control samples was 100%.

### Covariate assessment

Total dietary vitamin D, calcium and energy intake were assessed via validated frequency questionnaires as previously described (Calderon-Garcia et al., 2013; Moran et al., 2013).

### Statistical analysis

Allelic and genotypic frequencies were estimated by gene counting, and the goodness of fit of the genotype distribution for Hardy-Weinberg equilibrium (HWE) was tested by chi-square (*χ*^2^) test. Value of *P* > 0.05 indicated HWE.

Statistical analysis of the results was performed with SPSS 20 for Windows. Normal distribution and homogeneity of variances were assessed using the Kolmogorov–Smirnov and Levene tests, respectively. Analysis of variance (ANOVA) was used to compare different genotypes in each SNP followed by Bonferroni’s post-hoc test. Analysis of co-variance (ANCOVA) was used to compare the *VDR* genotypes adjusted for the co-variants age, BMI, years since menopause and daily calcium intake. A few variables were not normally distributed, and a bootstrap procedure was applied to the ANCOVA test. In that case, comparisons were performed with a non-parametric Kruskal Wallis test. Significance was set at *P* < 0.05.

## Results

The baseline characteristics of the 210 unrelated postmenopausal women are summarized in Table 1.

**Table 1:** Baseline characteristics of the studied sample.

	Mean	SD	Min	Max
Age (y)	60	8	42	78
Weight (kg)	62.9	10	42.6	92.3
Height (m)	1.54	0.06	1.39	1.68
BMI (kg/m^2^)	26.6	4.3	17.83	39.43
Menarche age (y)	12.9	1.45	9	16
Years since menopause (y)	13.1	8.8	0	38
Daily vitamin D intake (µg)	8.78	15.72	0.36	169.2
Daily calcium intake (mg)	1186.76	500.57	200	3169
Daily Kcal intake (kcalories)	2266	685.6	998.6	4452.8
BMD FN (g/cm^2^)	0.736	0.118	0.401	1.139
BMD FT (g/cm^2^)	0.579	0.107	0.268	0.955
BMD TW (g/cm^2^)	0.518	0.113	0.235	0.989
BMD L2 (g/cm^2^)	0.802	0.157	0.431	1.380
BMD L3 (g/cm^2^)	0.808	0.167	0.401	1.385
BMD L4 (g/cm^2^)	0.779	0.159	0.416	1.316
BMD L2-L4 (g/cm^2^)	0.796	0.156	0.418	1.310

The distribution of the BsmI genotype and alleles in the studied sample are shown in Table 2.

**Table 2:** Distribution of the BsmI restriction site genotypes and alleles in the studied sample.

	Genotype (%)			Allele		*P*
	Bb	bb	BB	b	B	
BsmI	45.2 (95/210)	41.4 (87/210)	13.3 (28/210)	0.6405	0.3595	0.79

Genotype frequencies at the two polymorphic sites agreed closely with Hardy-Weinberg ratios (*P* > 0.2). No baseline characteristic showed a significant difference between genotypes (Table 3).

**Table 3:** Distribution of baseline characteristics between the BsmI restriction site genotypes in the studied sample.

	BsmI vitamin D receptor genotype			
	bb (87)	Bb (95)	BB (28)	
	Mean ± SD	Mean ± SD	Mean ± SD	*P-value*
Age (y)	60 ± 8	60 ± 8	61 ± 9	0.78
Weight (kg)	61.9 ± 10	64.0 ± 9.9	62.1 ± 10.4	0.37
Height (m)	1.54 ± 0.05	1.54 ± 0.06	1.53 ± 0.06	0.86
BMI (kg/m^2^)	26.07 ± 4.02	27.10 ± 4.5	26.49 ± 4.41	0.34
Menarche age (y)	13.03 ± 1.44	12.79 ± 1.46	12.78 ± 1.47	0.48
Years since menopause (y)	12.8 ± 8.1	12.6 ± 9.1	15.7 ± 9.5	0.31
Daily vitamin D intake (µg)	8.15 ± 6.56	9.66 ± 2.17	7.62 ± 9.03	0.57
Daily calcium intake (mg)	1156.84 ± 496.88	1198.21 ± 428.25	1232.68 ± 705.52	0.61
Daily Kcal intake (kcalories)	2162.6 ± 574.6	2354.7 ± 756.2	2260.5 ± 710.1	0.4

The BsmI genotype was not related to BMD at any of the studied locations (all with *P* > 0.05 by ANOVA) (Table 4). A BMD analysis, after adjustment for potential confounding factors, such as age, BMI, and years since menopause, revealed no statistically significant differences between the genotypes (all with *P* > 0.05 by ANCOVA) (Table 4).

**Table 4:** BMD at the FN, FT, WT, L2, L3, L4 and L2-L4 according to the *VDR* BsmI. Data shown are mean ± SD, and *P* values obtained from ANOVA and ANCOVA. The BMD values are shown as BMD1 and BMD2, which denotes the raw BMD, and BMD adjusted for age, BMI, years since menopause and calcium intake respectively.

		BB (28)	Bb (95)	bb (87)	*P*-value		BB (28)	Bb (95)	bb (87)	*P*-value
BMD1 (g/cm^2^)	FN	0.739 ± 0.934	0.747 ± 0.129	0.725 ± 0.111	0.46	BMD2 (g/cm^2^)	0.723 ± 0.088	0.743 ± 0.122	0.713 ± 0.101	0.49
	FT	0.580 ± 0.090	0.586 ± 0.122	0.571 ± 0.094	0.61		0.569 ± 0.094	0.582 ± 0.112	0.558 ± 0.085	0.64
	TW	0.523 ± 0.087	0.525 ± 0.129	0.509 ± 0.101	0.61		0.506 ± 0.077	0.522 ± 0.119	0.504 ± 0.093	0.8
	L2	0.781 ± 0.131	0.819 ± 0.178	0.790 ± 0.138	0.33		0.762 ± 0.110	0.808 ± 0.166	0.778 ± 0.131	0.25
	L3	0.786 ± 0.131	0.931 ± 0.197	0.791 ± 0.136	0.2		0.764 ± 0.089	0.816 ± 0.187	0.776 ± 0.125	0.38
	L4	0.760 ± 0.130	0.796 ± 0.192	0.756 ± 0.125	0.34		0.748 ± 0.119	0.781 ± 0.185	0.755 ± 0.116	0.76
	L2-L4	0.775 ± 0.125	0.815 ± 0.187	0.783 ± 0.126	0.29		0.757 ± 0.099	0.799 ± 0.175	0.771 ± 0.115	0.63

**Notes.**

FN Femoral Neck FT Femoral trochanter TW Ward’s Triangle L2 L2 vertebra L3 L3 vertebra L4 L4 vertebra L2-L4 lumbar spine

Based on the FN T-score or the L2-L4 T-score, women were classified as normal (30 subjects, 14.3% of the study population), osteopenic (30 subjects, 14.3%), or osteoporotic (150 subjects, 71.4%) following the World Health Organization criteria (Kanis et al., 2002). The demographic, biological, major nutritional values and densitometric figures for osteoporotic, osteopenic and normal subjects are shown in Table 5. The osteoporotic women’s height, weight, and BMI were significantly lower than those of normal women (*P* < 0.05 by ANOVA). No statistically significant differences were found in age, menarche age, years since menopause, and daily intakes of calcium, vitamin D and kilocalories (*P* > 0.05 by ANOVA) (Table 5).

**Table 5:** Demographic, biological, major nutritional and densitometric figures for osteoporotic, osteopenic and normal subjects in the studied sample.

	NORMAL (30)		OSTEOPENIC (30)		OSTEOPOROTIC (150)		
	Mean	SD	Mean	SD	Mean	SD	*P*-value
Age	59.73	9.28	60.77	8.19	60.24	7.74	0.884
Menarche age	12.7	1.34	13.4	1.47	12.82	1.45	0.105
Years since menopause	12.76	8.23	13.37	9.31	13.08	8.8	0.968
Pregnancies	3	2	3	2	3	2	0.352
Weight (kg)	68.8	7.9	64.6	12	61.3	9.5	**0.001** ^a^
Height (m)	1.56	0.05	1.54	0.049	1.53	0.057	**0.041** ^b^
BMI (kg/m^2^)	28.29	3.9	37.34	5.39	26.1	4.051	**0.022** ^c^
Daily vitamin D intake (µg)	0.007	0.007	0.006	0.007	0.009	0.017	0.677
Daily calcium intake (mg)	1077.32	471.995	1240.269	559.722	1194.826	493.896	0.504
Daily Kcal intake (kcalories)	2084.52	626.98	2239	799.52	2302.68	670.52	0.38
BMD FN (g/cm^2^)	0.91	0.092	0.748	0.065	0.699	0.097	*P* < 0.0001
BMD FT (g/cm^2^)	0.736	0.087	0.577	0.083	0.548	0.085	*P* < 0.0001
BMD TW (g/cm^2^)	0. 682	0.101	0.529	0.075	0.483	0.09	*P* < 0.0001
BMD L2 (g/cm^2^)	1.124	0.092	0.806	0.047	0.737	0.085	*P* < 0.0001
BMD L3 (g/cm^2^)	0.154	0.126	0.796	0.092	0.742	0.076	*P* < 0.0001
BMD L4 (g/cm^2^)	1.115	0.096	0.803	0.042	0.706	0.073	*P* < 0.0001
BMD L2-L4 (g/cm^2^)	1.134	0.094	0.804	0.036	0.727	0.156	*P* < 0.0001

**Notes.**

a Normal vs Osteoporosis *P* = 0.01; Normal vs Osteopenia *P* = 0.298; Osteopenia vs Osteoporosis *P* = 0.282 by post hoc Bonferroni test.

b Normal vs Osteoporosis *P* = 0.035; Normal vs Osteopenia *P* = 0.363; Osteopenia vs Osteoporosis *P* = 1.000 by post hoc Bonferroni test.

c Normal vs Osteoporosis *P* = 0.032; Normal vs Osteopenia *P* = 1.000; Osteopenia vs Osteoporosis *P* = 0.437 by post hoc Bonferroni test.

The relationship between *VDR* gene genotypes and the BMD of osteoporotic, osteopenic and normal subjects is shown in Table 6. The BMD at L2 was lower in osteopenic women with the Bb genotype than in those carrying bb (*P* = 0.031), but after further adjustment by age, BMI and YSM, this difference was no longer statistically significant (*P* = 0.053).

**Table 6:** *VDR* BsmI genotype and BMD of osteoporotic, osteopenic and normal women.

	NORMAL				OSTEOPENIA				OSTEOPOROSIS			
	bb (8)	Bb (19)	BB (3)	*P*-value	bb (12)	Bb (11)	BB (7)	*P*-value	bb (67)	Bb (65)	BB (18)	*P*-value
DMO FN (g/cm^2^)	0.929 ± 0.089	0.910 ± 0.099	0.864 ± 0.006	0.295	0.739 ± 0.071	0.763 ± 0.058	0.740 ± 0.070	0.641	0.698 ± 0.093	0.696 ± 0.104	0.718 ± 0.095	0.552
DMO FT (g/cm^2^)	0.766 ± 0.111	0.735 ± 0.073	0.663 ± 0.088	0.224	0.556 ± 0.034	0.586 ± 0.122	0.603 ± 0.070	0.442	0.551 ± 0.071	0.544 ± 0.098	0.557 ± 0.091	0.722
DMO TW (g/cm^2^)	0.712 ± 0.093	0.676 ± 0.109	0.639 ± 0.060	0.47	0.513 ± 0.055	0.540 ± 0.105	0.540 ± 0.053	0.585	0.484 ± 0.080	0.479 ± 0.103	0.498 ± 0.087	0.821
DMO L2 (g/cm^2^)	1.147 ± 0.114	1.125 ± 0.086	1.060 ± 0.045	0.373	0.826 ± 0.040	0.778 ± 0.048	0.818 ± 0.041	**0.013** ^a^	0.741 ± 0.073	0.738 ± 0.097	0.721 ± 0.092	0.407
DMO L3 (g/cm^2^)	1.138 ± 0.109	1.169 ± 0.140	1.105 ± 0.079	0.777	0.782 ± 0.123	0.807 ± 0.084	0.805 ± 0.024	0.881	0.751 ± 0.065	0.737 ± 0.088	0.727 ± 0.069	0.316
DMO L4 (g/cm^2^)	1.09 ± 0.094	1.132 ± 0.097	1.055 ± 0.090	0.352	0.803 ± 0.020	0.800 ± 0.067	0.810 ± 0.016	0.778	0.719 ± 0.060	0.698 ± 0.086	0.691 ± 0.066	0.393
DMO L2-L4 (g/cm^2^)	1.127 ± 0.097	1.146 ± 0.099	1.074 ± 0.026	0.355	0.814 ± 0.012	0.792 ± 0.057	0.811 ± 0.011	0.461	0.737 ± 0.055	0.722 ± 0.082	0.711 ± 0.064	0.355

**Notes.**

a After further adjustment by potential confounding factors (age, BMI, years since menopause and calcium intake) (Bootstrap ANCOVA test) *P* = 0.053.

## Discussion

Genetic association studies in osteoporosis often bring discrepant results. Our study found no direct association between BMD and specific genotypes of the analyzed *VDR* polymorphism. Our results are also consistent with previous studies, (Uitterlinden et al., 1996; Fang et al., 2006; Horst-Sikorska et al., 2013) in which no relationship between *VDR* polymorphisms BsmI, ApaI or other (TaqI and FoqI) and BMD was found. Moreover, in postmenopausal women undergoing antiresorptive treatment, therapy response was independent of the BsmI genotype (Conti et al., 2015). However, a meta-analysis by Thakkinstian and co-workers (2004) revealed a weak but statistically significant association between the B allele and lower BMD in the lumbar spine; these results support a former paper from Morrison et al. (1994) that reported lower BMD in BsmI BB homozygotes. Recently, a meta-analysis from Wang and colleagues (2013) found an association of BMD with BsmI and ApaI polymorphisms in postmenopausal Asian women (especially Indian women), suggesting that the racial/ethnic genetic background plays a role. In addition, Jia and colleagues (2013) recently found an association of the *VDR* BsmI polymorphism with a protective role against the development of osteoporosis in postmenopausal women using a meta-analysis of 26 studies. At this point, studies have reported controversial results, and no conclusion about the role of common *VDR* polymorphisms on BMD can be drawn yet.

Hence, discordant results may be due to the different populations studied/racial differences in BMD. A study by Morrison et al. (1994) was conducted among an ethnically diverse Australian population, which was additionally exposed to higher doses of UVB radiation and potentially higher amounts of active vitamin D.

*VDR* gene expression is likely affected by environmental factors. Several authors suggested that calcium homeostasis might play a role in this process. Stathopoulou and colleagues (2011) showed that, under lower calcium intake (<680 mg/day), the presence of the B allele of the BsmI polymorphism increased the risk of osteoporosis by 118%. In the presence of higher calcium intake, the influence of the *VDR* alleles on BMD was nearly insignificant. Thus, adequate calcium intake appears to counteract the genetic influence of *VDR* on the bone, and this effect that could be observed in our study. In fact, participants in our study had a daily calcium intake of 1186.76 mg, which was almost double the amount reported by Stathopoulou and colleagues (2011). We hypothesize that these findings further support the hypothesis that environmental factors, especially dietary calcium, may modulate the genotype-phenotype relationship, and therefore have a significant impact on the obtained results.

Thus, an important environmental-gene interaction exists between *VDR* polymorphisms, including BsmI variants, and calcium or calcitriol intake. The first evidence for an interaction between calcium intake and the BsmI *VDR* polymorphism came from a study by Ferrari et al. (1995) in a sample of elderly patients receiving calcium and calcitriol supplements, among whom BMD loss was higher in the lumbar spine of the BB homozygotes. Additionally, the BB genotype is associated with a higher response to calcium administration, reduced efficiency of calcium absorption and low response of BMD (Gennari et al., 1997). Furthermore, a large-scale population-based study from MacDonald and colleagues (2006) reported evidence of weak interactions between calcium intake, *VDR* variants and BMD with rates of bone loss in a manner that favored the bb homozygotes in the lowest calcium intake quartile.

Additionally, the BsmI polymorphism is located in the non-coding region of the *VDR* gene, and they do not have an effect on the final protein product (Uitterlinden et al., 2004; Fang et al., 2005). The B allele correlate with enhanced mRNA stability or transcriptional activity and greater vitamin D activity (Morrison et al., 1994). Overall, this fact highlights the importance of understanding the mechanisms by which these polymorphisms affect *VDR* action.

In Spain, 719 postmenopausal women were genotyped for common *VDR* polymorphisms (Bustamante et al., 2007), and a lack of relevance for osteoporosis and these polymorphisms was found. Our results agree with this much larger study in Spanish postmenopausal women. In a cohort of 177 Spanish postmenopausal women, Bandres and colleagues (2005) did not find an association of BMD with the BsmI gene polymorphism after further adjustment for potential confounding factors (weight, age and years since menopause). Similar results were found in a smaller study from Fontova Garrofé and colleagues (2000) (for the BsmI polymorphism). However, in a cohort of 204 Spanish postmenopausal women, a significantly lower BMD was observed in BB subjects (Bernad et al., 1999). Interestingly, 60% of the studied sample had an intake of calcium lower than 500 mg/day, and these numbers are in the range reported by Stathopoulou and colleagues (2011). This result could support the reported association because the calcium intake would not mask the *VDR* genetic influence on bone in the study by Bernard and colleagues.

Our study was limited by the modest sample size, which did not have sufficient statistical power for the detection of subtle effects; therefore, there might be type II errors.

## Conclusions

In conclusion, we found no significant association between the common *VDR* polymorphism BsmI and BMD at any skeletal site in a sample of Spanish postmenopausal women. Previous studies in Spain have examined the association between BMD and the *VDR* polymorphism BsmI, and to date, our study is one of the highest in sample size. The results reported should be interpreted cautiously because of the small sample size. Future studies in larger samples of Spanish postmenopausal women focused in the study of the different haplotypes, instead of a single polymorphism will help to clarify potential effects of common *VDR* polymorphisms.

## Supplemental Information

10.7717/peerj.953/supp-1 Data S1 Raw data
